# Evidence for Oxidative Stress and Defective Antioxidant Response in Guinea Pigs with Tuberculosis

**DOI:** 10.1371/journal.pone.0026254

**Published:** 2011-10-18

**Authors:** Gopinath S. Palanisamy, Natalie M. Kirk, David F. Ackart, Crystal A. Shanley, Ian M. Orme, Randall J. Basaraba

**Affiliations:** Department of Microbiology, Immunology and Pathology, Colorado State University, Fort Collins, Colorado, United States of America; Hopital Raymond Poincare - Universite Versailles St. Quentin, France

## Abstract

The development of granulomatous inflammation with caseous necrosis is an important but poorly understood manifestation of tuberculosis in humans and some animal models. In this study we measured the byproducts of oxidative stress in granulomatous lesions as well as the systemic antioxidant capacity of BCG vaccinated and non-vaccinated guinea pigs experimentally infected with *Mycobacterium tuberculosis*. In non-vaccinated guinea pigs, oxidative stress was evident within 2 weeks of infection as measured by a decrease in the serum total antioxidant capacity and blood glutathione levels accompanied by an increase in malondialdehyde, a byproduct of lipid peroxidation, within lesions. Despite a decrease in total and reduced blood glutathione concentrations, there was an increase in lesion glutathione by immunohistochemistry in response to localized oxidative stress. In addition there was an increase in the expression of the host transcription factor nuclear erythroid 2 p45-related factor 2 (Nrf2), which regulates several protein and non-proteins antioxidants, including glutathione. Despite the increase in cytoplasmic expression of Nrf2, immunohistochemical staining revealed a defect in Nrf2 nuclear translocation within granulomatous lesions as well as a decrease in the expression of the Nrf2-regulated antioxidant protein NQO1. Treating *M. tuberculosis*–infected guinea pigs with the antioxidant drug N-acetyl cysteine (NAC) partially restored blood glutathione concentrations and the serum total antioxidant capacity. Treatment with NAC also decreased spleen bacterial counts, as well as decreased the lung and spleen lesion burden and the severity of lesion necrosis. These data suggest that the progressive oxidative stress during experimental tuberculosis in guinea pigs is due in part to a defect in host antioxidant defenses, which, we show here, can be partially restored with antioxidant treatment. These data suggest that the therapeutic strategies that reduce oxidant-mediated tissue damage may be beneficial as an adjunct therapy in the treatment and prevention of tuberculosis in humans.

## Introduction

Infections with *Mycobacterium tuberculosis*, the causative agent of human tuberculosis, is the most common cause of morbidity and mortality in patients with HIV/AIDS and is among the leading causes of death from an infectious agent [1]. The continuing spread of tuberculosis is evident from the 2009 figures that estimate the number of newly diagnosed cases of active tuberculosis worldwide to be 9.4 million [2]. Controlling the spread of tuberculosis, particularly in developing countries, has become more challenging by the emergence of multidrug (MDR) and extensively drug-resistant (XDR) strains of bacilli that are refractory to first and second line anti-tuberculosis drugs respectively [3]. While several promising new drugs are in early clinical trials, no new FDA approved anti-tuberculosis drugs have been introduced in the last 25 years. Moreover, the effective treatment of patients for 6 to 9 months with existing drugs is limited in some patients by the complications associated with toxic side effects of combination drug therapy. Development of new anti-tuberculosis drugs or treatment strategies that potentiate the therapeutic value of currently available drugs is urgently needed for global tuberculosis control.

 The difficulty in effectively treating human tuberculosis is related to the chronic nature of the disease and the long-term persistence of drug-tolerant bacilli. There is increasing evidence from human and animal studies that at least one population of drug-tolerant bacilli persist within an extracellular microenvironment associated with necrotic and cavitary lesions [4], [5]. A better understanding of the pathogenesis of lesion progression may reveal novel therapeutic approaches that prevent or specifically target bacilli that persist within this unique *in vivo* microenvironment. Tuberculosis, like other chronic inflammatory diseases, is characterized by the generation of oxygen and nitrogen free radicals. The generation of free radicals in excess of the antioxidant capacity of the host leads to cellular and eventually systemic oxidative stress. Markers of oxidant-mediated tissue damage have been shown to be elevated in the peripheral circulation of humans with active tuberculosis [6], [7]. Moreover, oxidative stress has been implicated in the pathogenesis of lung fibrosis and dysfunction in tuberculosis patients even following antimicrobial therapy [6], [8]. Besides an increase in the byproducts of free radical generation, several studies have demonstrated that critical antioxidants such as ascorbic acid and glutathione are depleted in the serum of tuberculosis patients [9], [10]. Oxidative stress resulting from the consumptive depletion of protein and non-protein antioxidants can be further complicated by inadequate dietary replenishment [9], [11].

Besides vaccination and combination antimicrobial therapy, few interventions have been shown to be effective at reversing or preventing the progression of active tuberculosis in humans or animals. Previous studies have shown that in the guinea pig tuberculosis model, the progression of pulmonary and extra-pulmonary lesions can be modulated by vaccination with Bacillus Calmette Guerin (BCG) prior to aerosol infection with virulent *M. tuberculosis* [12], [13]. The partially protective immune response primed by BCG vaccination lessens the severity of disease, delays extra-pulmonary dissemination of bacilli and prevents the progressive accumulation of free radical generating leukocytes including macrophages and granulocytes [14].

The collective antioxidant capacity of the host is maintained by numerous intracellular and circulating protein and non-protein molecules, which prevent oxidative damage to lipids, proteins, and nucleic acids. The group of protein antioxidants that detoxify free radicals through conjugation are referred to as phase II detoxification enzymes [15]. The induction of phase II enzyme expression requires binding of specific inducers to the antioxidant response element (ARE) in the promoter regions of phase II enzyme genes. Nuclear erythroid 2 p45-related factor 2 (Nrf2), a redox-sensitive transcription factor, is directly involved in the transcriptional activation of ARE-driven phase II antioxidant enzymes. Under normal physiologic conditions, Nrf2 is sequestered in the cytoplasm by the binding protein, Kelch-like ECH-associated protein (Keap1). Under oxidative stress conditions, Nrf2 dissociates from Keap1 allowing translocation of Nrf2 to the nucleus where it heterodimerizes with small Maf-family proteins which bind to ARE sequences leading to transcriptional activation of the various phase II antioxidant enzymes [16].

NAD(P)H dehydrogenase, quinone 1 (NQO1) is one such protein that prevents free radical formation from quinone biosynthesis. Glutathione, another Nrf2-regulated antioxidant is an essential intracellular antioxidant tripeptide that is synthesized within cells in a two-step, energy dependent reaction. The first and rate-limiting step in the synthesis of glutathione is catalyzed by glutamate-cysteine-ligase (GCL; also known as γ-glutamylcysteine synthetase-GCS), which is also transcriptionally regulated by Nrf2 [17], [18].

The goals of these studies was to determine what role oxidative stress has in the progression of experimental tuberculosis in the guinea pig and whether antioxidant therapy was effective at lessening the bacterial burden and disease severity. Additionally, we sought to determine whether a defect in antioxidant defenses, particularly those regulated by Nrf2 could be restored with the antioxidant drug N-acetyl cysteine. We also asked whether the reduction in disease severity in *M. tuberculosis* infected guinea pigs that were BCG vaccinated prior to challenge was due to preservation of antioxidant capacity and Nrf2 function. Our data suggest that antioxidant therapy may prove beneficial as an adjunct therapy to reduce disease severity by limiting host tissue damage. These data suggest that antioxidant therapy could be used to potentiate the effectiveness of conventional prevention and treatment strategies in human patients with tuberculosis.

## Methods

### Aerosol infection of guinea pigs with *M. tuberculosis*

*Mycobacterium tuberculosis* H37Rv strain (TMC#102; Trudeau Institute, Saranac Lake, NY) was grown in Proskauer-Beck liquid medium containing 0.05% Tween 80 to mid-log phase, aliquoted, and frozen at −80°C until used for infection. A thawed aliquot of *M. tuberculosis* was diluted in sterile water to 10^6^ CFU/ml for a total working stock volume of 20 ml. Guinea pigs (approximately 9 months of age) from each treatment group were aerosolized using the Madison infection chamber (University of Wisconsin Machine Shop, Madison, WI) with a starting volume of 15 ml of working stock [19].

### BCG vaccination

Guinea pigs were vaccinated with 1×10^4^ *Mycobacterium bovis* (BCG, strain Pasteur) or mock vaccinated with equal volumes of saline, intra-dermally 4 weeks prior to aerosol exposure to *M. tuberculosis*. *M. bovis* BCG was grown in Proskauer-Beck similar to *M. tuberculosis* and frozen at −80°C until used for vaccination [14].

### N-acetyl cysteine treatment

Guinea pigs were treated or mock-treated orally with N-acetyl cysteine (NAC) (400 mg/kg, Calbiochem, San Diego, CA) from day 0 to the experimental end-point (day 30 or 60 of infection) dissolved in 2 ml water containing 20% sucrose or carrier alone.

### Euthanasia and sample collection

At intervals of 5, 15, 20, 30 and 60 days of infection, guinea pigs were euthanized humanely by an overdose (1 ml per 0.75 kg body weight) of sodium pentobarbital (Sleepaway; Fort Dodge Laboratories Inc.) by intraperitoneal injection. Following euthanasia, at each time point, the left pulmonary lobes were infused *in situ* and fixed for 48 hours in 4% paraformaldehyde and thereafter stored in 70% ethanol. At the time of processing, all tissues were embedded in paraffin, sectioned at 5 µm, and stained with hematoxylin and eosin (H&E). At the same time points, bacterial load of lungs, spleen and peribronchial lymph nodes were determined by plating respective organ homogenates onto nutrient 7H11 agar plates supplemented with OADC. Colonies are counted after 21 days of incubation at 37°C [19].

### Measurement of serum total antioxidant capacity and blood glutathione levels

Serum from guinea pigs was assayed for total antioxidant capacity (Sigma, St. Louis, MO), and whole blood to determine the oxidized/reduced-glutathione levels and GSH/GSSG ratio (GSH/GSSG Ratio Assay kit; Calbiochem, San Diego, CA) using calorimetric assays according to the manufacturer's protocols.

### Immunohistochemistry

Paraffin embedded sections of lung from the experimentally infected guinea pigs, were collected on positively charged glass slides, deparaffinized, rehydrated and subjected to antigen retrieval by incubation in Target Retrieval solution, pH 6.0 (DAKO, Carpentaria, CA) for 25 min at 90°C, followed by a 20 min cooling period at room temperature. The sections were then treated with 0.3% hydrogen peroxide in water for 15 min to quench endogenous peroxidase activity. Following a rinse in Tris buffered saline with 1% Tween-20 (TTBS), the slides were subjected to two blocking steps: (i) 15 min incubation with 0.15 mM glycine in PBS, and (ii) 30 min incubation with 1% normal horse serum with a rinse in TTBS in between. The slides were then incubated with rabbit polyclonal antibody to human Nrf2, GCS (Santa Cruz Biotechnology, Santa Cruz, CA), MDA, NQO1 (Abcam, Cambridge, MA) or mouse monoclonal antibody against glutathione (Virogen, Watertown, MA) or respective isotype IgG controls at a 1∶100 dilution in blocking buffer followed by several rinses in TTBS. This was followed by 30 min incubation with biotinylated goat-anti rabbit-IgG (Vector Laboratories) and visualization of bound antibody by the Avidin-Biotin system (Vectastain; Vector Laboratories) and diaminobenzidine substrate (Dako; Carpentaria, CA). The sections were counterstained with Meyer's hematoxylin (Scytek Laboratories; Logan, Utah), mounted with coverslips, and examined on an Olympus BX41 light microscope.

The specificity of anti-human antibodies for guinea pig Nrf2 and NQO1was validated by competitive inhibition of binding to the tissue sections with purified antigens (Nrf2 - Santa Cruz Biotechnology; NQO1 - Abcam) at a concentration ratio of 1∶5 before adding the primary antibody to the respective sections in lieu of the unblocked primary antibodies. Antibody specificity was confirmed by the effective blocking of anti–human Nrf2 and NQO1 antibodies binding to guinea pig tissues with the respective purified proteins (Figure S1). Photomicrographs were acquired with an Olympus DP70 camera and the associated computer software. The reviewer blinded to the treatment groups scored sections stained by H&E and immunohistochemistry as described below.

### Immunohistochemical scoring

The lung lesions were classified as either primary lesions or primary lesion free (PLF) lung for immunohistochemical scoring purposes. Primary lung lesions are the initial foci of inflammation that develop following aerosol infection of immunologically naïve animals and are characterized by central necrosis. Secondary lesions are the foci of inflammation that develop in the late stage of infection as a consequence of hematogenous dissemination. Primary lesion free lung constitutes non-necrotic secondary lesions and residual normal lung parenchyma [20]. Within these regions the overall immunohistochemical scoring was determined based on extent of staining (0-none; 1-less than 25%; 2-26–50%; 3-51–75%; 4-more than 75%) and staining intensity (0-none: 1-mild; 2-moderate; 3-marked; 4-extensive). The resultant total overall scores were converted to a four-point scale. Since the *in vivo* function of Nrf2 as a transcription factor is dependent on cytoplasmic to nuclear translocation, the relative nuclear and cytoplasmic expression of Nrf2 in lesions and PLF lung was also scored using the following scale (0-none; 1-less than 25%; 2-26–50%; 3-51–75%; 4-more than 75%).

### Lesion analysis

To evaluate the progression of lesions over time, a histological grading system was applied, as previously described [21]. The lung sections were scored based on the following six criteria: (i) percent of lung affected: 0-no lesions in lung, 1-up to 25% of lung involved, 2-up to 50% of lung involved, 3-up to 75% of lung involved, 4-above 75% of lung involved. (ii) primary lesions: 0-no primary lesions present, 1-a single primary lesion, 2-two or more primary lesions, multi-focal, 3-two or more primary lesions, multifocal to coalescing, 4-multiple primary lesions, coalescing and extensive. (iii) secondary lesions: 0-no secondary lesions present, 1-up to 25% of lung involved, 2-up to 50% of lung involved, 3-up to 75% of lung involved, 4-above 75% of lung involved. Necrosis (iv), mineralization (v) and fibrosis (vi) are scored based on severity as follows: 0-none, 1-minimal, 2-mild, 3-moderate, 4-marked. The subcategory scores were added for the final total score for each organ (Range: 0–24). The spleen lesions were scored based on four categories; (i) percentage involvement (same scale as lungs), (ii) degree of necrosis, (iii) fibrosis and (iv) mineralization. The total spleen score range is 0 to 16.

### Statistical analysis

The statistical differences of lesion/necrosis scores or immunostaining scores among different time points were analyzed using non-parametric Kruskal-Wallis test. The specific statistical differences between two individual group scores were analyzed using non-parametric Mann-Whitney test. The statistical differences between numbers of bacilli, antioxidant levels, total glutathione levels and GSH/GSSG ratio were analyzed using two-way ANOVA and non-paired student t test. Statistical analysis of correlation was performed for the groups discussed in the results. All of the statistical tests were done using the Prism software (version 4.03, Graph Pad, La Jolla, CA).

## Results

### Presence of malondialdehyde (MDA) in infected guinea pig lungs

**Figure 1:**
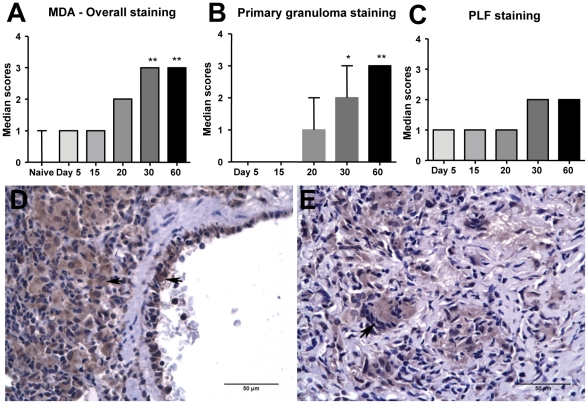
There is increased malondialdehyde accumulation in the lungs of *M. tuberculosis* infected guinea pigs. The graphs A, B and C represent MDA immunostaining scores within overall, primary granuloma and primary lesion free (PLF) lung areas respectively at different time points of Mtb H37Rv infection (median+range, n = 5). The stars denote statistically significant increase compared to the naive animals (* = p<0.05 and ** = p<0.01). The photomicrographs D and E represent immunostaining of MDA in Mtb-infected guinea pig lungs from day 30 and day 60 of infection respectively (arrowheads show intracellular staining within macrophages and vascular endothelial cells).

MDA is one of the major aldehyde byproducts of lipid peroxidation that accumulate in the peripheral circulation and in tissues under oxidative stress conditions. During the generation of reactive oxygen species (ROS), MDA forms numerous protein adducts that can be specifically detected by immunohistochemistry. There was a statistically significant (p<0.05) increase in MDA expression in *M. tuberculosis*-infected guinea pig lungs beginning on day 20 of infection that progressed between days 30 and 60 (Figure 1A). Lungs from *M. tuberculosis*-infected guinea pigs on day 5 of infection and non-infected animals showed minimal or no immunostaining for MDA. The accumulation of MDA showed a strong positive correlation with pulmonary lesion progression (r = 0.97 and p≤0.005; lesion scores not shown). The progressive increase in MDA expression was predominantly within the primary granulomas (Figure 1B). Overall immunostaining scores represented the sum total scores for both primary lesions and primary lesion-free (PLF) lung. The staining was comparatively unchanged within the PLF lung at different time points (Figure 1C). The cell types that showed the most intense MDA staining included macrophages, vascular endothelial cells (Figures 1D and E) and granulocytes.

### Serum total antioxidant capacity

**Figure 2:**
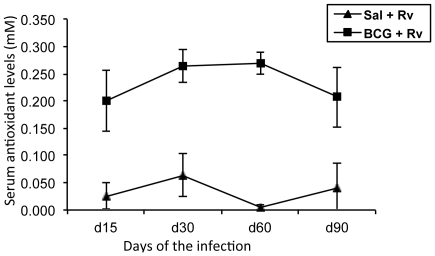
*M. tuberculosis* infection of guinea pigs results in decreased serum antioxidant capacity. The graph illustrates systemic mean (± SD) total antioxidant levels in serum of Mtb-infected guinea pigs that are either saline vaccinated (control) or BCG vaccinated (n = 5). The mean total antioxidant level in serum of naïve guinea pigs is 0.211±0.042 mM (n = 5).

The total antioxidant capacity of the serum was determined using an assay based on the ability of serum antioxidants to quench radical cation (soluble chromogen) production when ferryl myoglobin reacts with an enzyme substrate. Total antioxidant capacity of the serum samples collected from mock-vaccinated and BCG- vaccinated-*M. tuberculosis*-infected guinea pigs on days 15, 30, 60 and 90 of infection are illustrated in Figure 2. The serum total antioxidant capacity in *M. tuberculosis*-infected guinea pigs mock-vaccinated with saline ranged from undetectable levels as early as 15 days of infection to 0.06±0.06 mM, whereas the total antioxidant levels in the BCG-vaccinated, *M. tuberculosis* infected animals were four to five fold higher ranging from 0.20±0.06 mM to 0.27±0.04 mM. The antioxidant capacity of naïve un-infected guinea pigs was similar to that of BCG vaccinated guinea pig values being 0.211±0.042 mM (n = 7). There was no statistical difference in antioxidant levels between uninfected naive guinea pigs and BCG vaccinated infected animals and BCG vaccination normalizes systemic antioxidant levels to uninfected values.

### Whole blood glutathione levels

**Figure 3:**
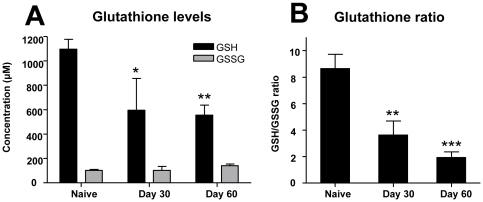
Total and reduced blood glutathione levels are decreased during *M. tuberculosis* infection in guinea pigs. The graph A denotes the systemic levels of reduced- and oxidized-glutathione (GSH and GSSG respectively) in the blood of naive and Mtb-infected guinea pigs on days 30 and 60 after the infection. The graph B illustrates the ratio between reduced- and oxidized-glutathione in blood from the same animals. Stars represent statistically significant decrease compared to the naive animals (* = p<0.05, ** = p<0.01 and *** = p<0.001).

Glutathione, a thiol tripeptide antioxidant is among the most critical antioxidants and is present in the reduced (GSH) and oxidized form (GSSG) in all mammalian cells [22]. *M. tuberculosis* infection in guinea pigs resulted in a significant reduction in the reduced-glutathione levels in whole blood on days 30 and 60 after infection compared to the naive animals (Figure 3A). The oxidized-glutathione levels did not change significantly throughout the course of infection (Figure 3A). However, there was a progressive decrease in the blood antioxidant capacity as expressed as a ratio of reduced- and oxidized-glutathione (GSH/GSSG) (Figure 3B).

### Glutathione expression in lung lesions

**Figure 4:**
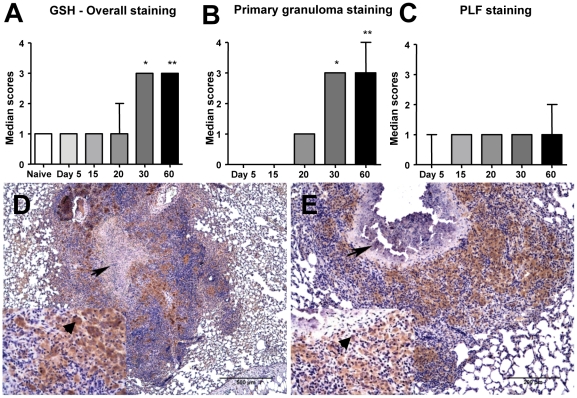
Glutathione expression is increased in lung lesions of *M. tuberculosis-*infected guinea pigs. The graphs A, B and C represent glutathione immunostaining scores (median+range, n = 5) within overall, primary granuloma and primary lesion free (PLF) lung areas respectively at different time points (* = p<0.05 and ** = p<0.01 when compared to the naive animals). Figures D and E illustrate predominant glutathione staining in the primary granulomas (arrows) with necrotic core on day 30 (non-mineralized core) and on day 60 (mineralized core). Inserts show the intracellular staining within the macrophages (200×, arrowheads).

Tissue glutathione expression was estimated in the lungs from *M. tuberculosis*-infected guinea pigs by immunohistochemistry. There was a statistically significant (p<0.05) increase in expression of glutathione in lung lesions from *M. tuberculosis*-infected guinea pig on days 30 and 60 (Figure 4A). Similar to MDA, the increase in glutathione immunostaining occurred predominantly within the primary granulomas (Figure 4B) and less so in the PLF lung across the different time points (Figure 4C). The pattern of lung cell glutathione expression was similar to that of MDA (Figures 4D and E).

### Nrf2 expression in lungs

**Figure 5:**
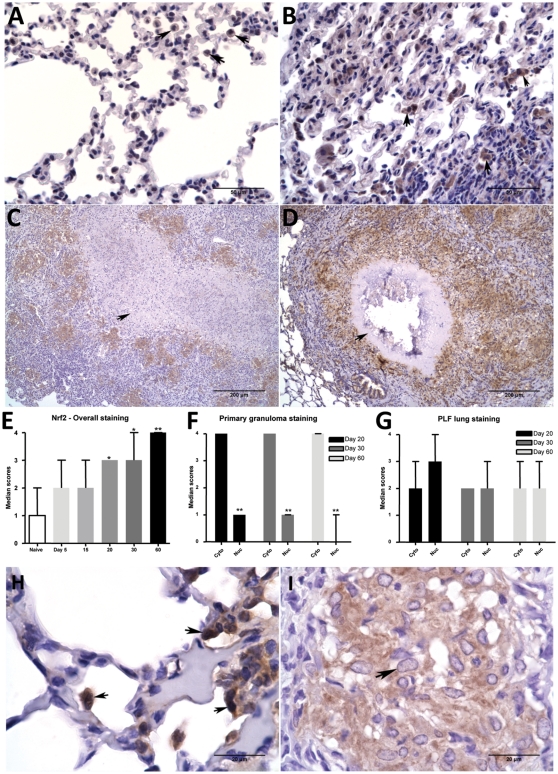
Intracellular Nrf2 expression is increased in lung lesions of *M. tuberculosis* infected guinea pigs. The photomicrographs A, B, C and D represent Nrf2 immunostaining within infected lungs from day 0, 20, 30 and 60 respectively. Arrowheads (in A&B) show intracellular staining within macrophages. Arrows (in C & D) show primary granulomas with necrotic core demonstrating Nrf2 staining. The graph E represents Nrf2 overall lung immunostaining scores at different time points of infection. The bars represent median values (+range) for each group (n = 5). The stars denote statistically significant increase compared to the naive animals (* = p<0.05 and ** = p<0.01). The graphs F and G represent median cytoplasmic and nuclear staining scores (+range) at different time points within primary granuloma and PLF lung areas respectively. The photomicrographs H and I represent immunostaining of Nrf2 in PLF lung (arrows show both intracytoplasmic and nuclear staining within macrophages) and primary granuloma (arrowhead shows predominantly intracytoplasmic but no nuclear staining of Nrf2 in macrophages) of a same animal on day 30.

Nrf2 is a crucial redox sensitive transcription factor that controls expression of several important antioxidant enzymes including NQO1 and GCS. There was an overall increase in Nrf2 immunostaining in *M. tuberculosis*-infected guinea pig lung lesions as the infection progressed (Figures 5A through 5E). Nrf2 staining was predominant within the primary granulomas on days 30 and 60 (Figures 5C and 5D). Closer examination using higher magnification revealed roughly equal cytoplasmic and nuclear expression of Nrf2 throughout the lungs early in the infection (days 5 and 15) and in less-affected PLF regions of lungs late in the infection (days 30 and 60) (Figures 5G and 5H). In contrast, within epithelioid macrophages associated with primary lesions, expression of Nrf2 was predominantly cytoplasmic with minimal nuclear staining (p<0.05) as early as day 20, which was pronounced by day 30 and 60 (Figures 5F and 5I).

A similar lack of nuclear expression of Nrf2 was seen in guinea pig bronchoalveolar lavage (BAL) cells at 4, 24, 48 and 72 hrs after *in vitro* infection with *M. tuberculosis* (Figure S2). Only a weak cytoplasmic and no nuclear staining of Nrf2 was evident starting as early as 4 hours after infection.

### Nrf2-regulated antioxidant protein expression

**Figure 6:**
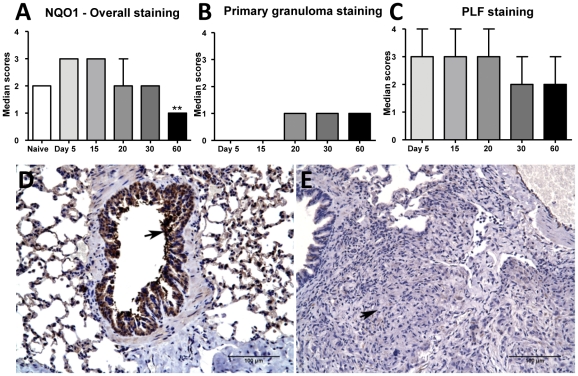
Nrf2-regulated antioxidant protein NQO1 expression is decreased in lung lesions from *M. tuberculosis* infected guinea pigs. The graphs A, B and C represent NQO1 immunostaining scores within overall, primary granulomas and primary lesion free (PLF) lung areas respectively at different time points. The bars represent median values (+range) for each group (n = 5). The photomicrographs D and E represent immunostaining of NQO1 in *M. tuberculosis* -infected guinea pig lungs on day 5 (arrow shows strong immunoreactivity within airway epithelial cells) and day 30 (arrow points to a granuloma with no immunoreactivity) respectively.

The lack of Nrf2 nuclear translocation suggesting a defect in Nrf2-mediated antioxidant defenses was confirmed by evaluating the expression of the Nrf2-regulated expression of NQO1 and GCS. NQO1 expression decreased as the infection progressed (Figure 6A) which inversely correlated with increased Nrf2 cytoplasmic expression over time (r = −0.96). Throughout the infection, immunostaining of NQO1 was localized within the PLF regions of the lungs where NQO1 expression overlapped with the high Nrf2 expression levels (Figures 6C). The primary granulomas on day 20, 30 and 60 showed minimal NQO1 expression (Figures 6B and 6E). Airway epithelium (Figure 6D), vascular endothelium and alveolar macrophages consistently expressed NQO1 in the early stages of infection (days 5, 15 and 20). As the disease progressed, only minimal immunostaining of these cells was seen (Figure 6E).

The expression of GCS was less pronounced than NQO1 in the lungs of infected guinea pigs compared to non-infected in naïve guinea pig lungs. However, no difference in GCS immunostaining was seen between days 5 and 60. Also, no significant difference in expression levels of GCS was observed between primary granuloma and PLF lungs (data not shown).

### Effect of NAC treatment of *M. tuberculosis* infected guinea pigs

**Figure 7:**
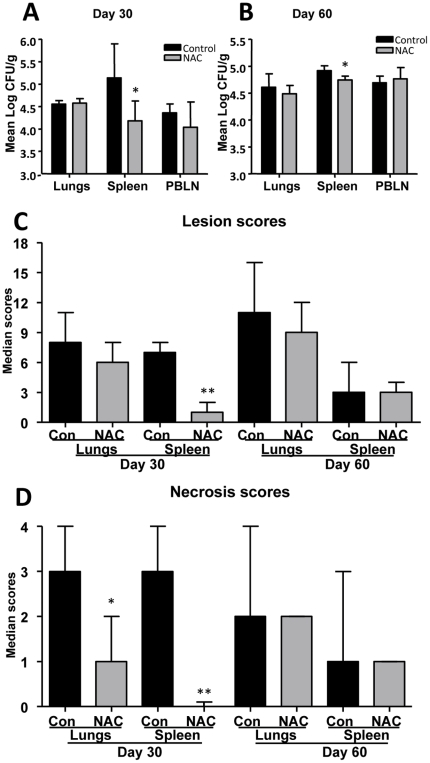
Treatment of *M. tuberculosis* infected guinea pigs with NAC reduces the bacterial burden and extra-pulmonary disease severity. The graphs A and B represent mean numbers of bacilli in different organs from control and NAC treated guinea pigs on day 30 and 60 respectively (mean+SD, n = 5). The graphs C and D represent overall lesion and necrosis scores (median+range) respectively of lungs and spleen from control and NAC treated animals on days 30 and 60 (n = 5). Statistically significant change, if present, is indicated with stars (** = p<0.01).

As NAC has been shown to induce Nrf2-mediated antioxidant defenses, *M. tuberculosis*-infected guinea pigs were treated with NAC in an attempt to reverse the adverse effects of oxidative stress [23]. The daily administration of NAC resulted in nearly one log reduction in the number of bacilli in the spleen on day 30 which correlated with a significant decrease in lesion burden (Figure 7A and 7C) which was less but still statistically significant by day 60 (Figures 7B). No significant differences in the numbers of bacilli were observed between control and NAC-treated groups on day 30 and 60 in lungs and peribronchial lymph node (PBLN) (Figures 7A and 7B).

**Figure 8:**
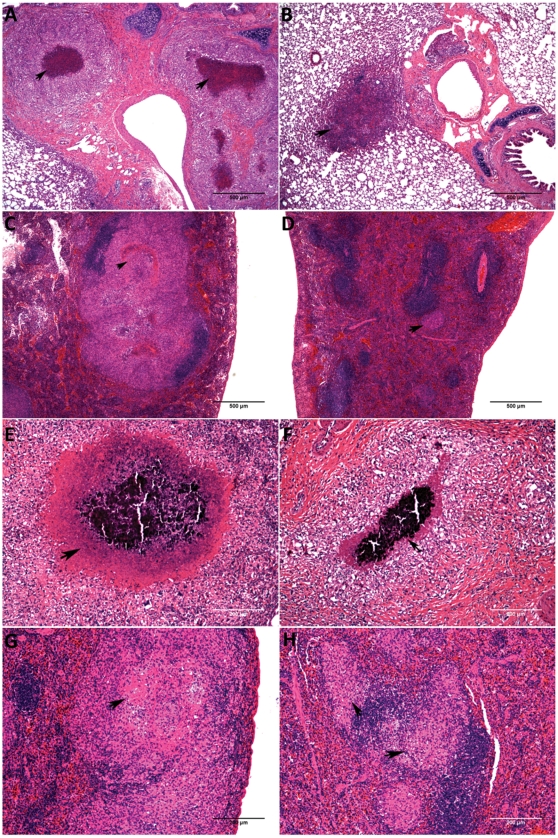
Treatment of *M. tuberculosis* infected guinea pigs with NAC reduces pulmonary and extra-pulmonary granuloma necrosis. The photomicrographs A, B, C and D represent lesion pathology in the lungs (A & B) and spleen (C & D) on day 30 in control (A & C) and NAC (B & D) treated animals. The arrows in A and C (controls) show a large core of necrosis and the arrowheads in B and C (NAC treated) point to granulomas with minimal to no necrosis. The photomicrographs E, F, G and H represent the lesion burden in the lungs (E & F) and spleen (G & H) on day 60 in control (E & G) and NAC (F & H) treated animals. The arrows in E and G (controls) show a large area of necrosis and the arrowheads in F and H (NAC treated) show the granulomas with smaller area of necrosis.

A statistically significant reduction in overall spleen lesion scores (p<0.01) in NAC-treated animals was observed on day 30 of infection (Figure 7C). Moreover, statistically significant reduction in lesion necrosis scores was seen in NAC-treated guinea pig lungs (p<0.05) and spleen (p<0.01) compared to the control groups on day 30 of the infection (Figures 7D). The decrease in lesion burden and extent of necrosis in the lungs and spleen of NAC-treated animals are depicted in Figure 8 (A, B, E & F-lungs; C, D, G & H-spleen).

**Figure 9:**
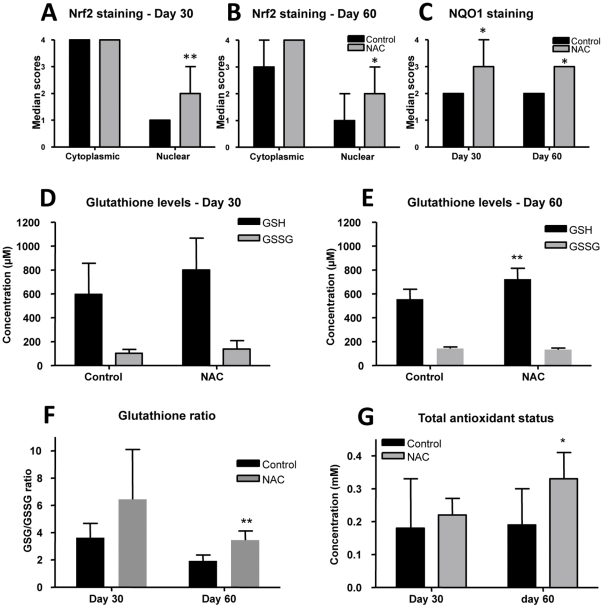
NAC treatment of *M. tuberculosis* infected guinea pig partially restores antioxidant capacity. The graphs A and B represent the cytoplasmic and nuclear Nrf2 staining scores within primary granulomas of animals treated with control or NAC on days 30 and 60 respectively (median+range, n = 5). The graph C represents NQO1 staining within primary granulomas of animals treated with control or NAC on days 30 and 60 (median+range, n = 5). The graphs D and E represent reduced- and oxidized-glutathione levels (GSH and GSSG) in control and NAC treated animals on days 30 and 60 respectively (mean+SD, n = 5). The graph F represents GSH/GSSG ratio in the blood of control or NAC-treated guinea pigs on days 30 and 60. The graph G represents the systemic total antioxidant levels of serum from control and NAC treated guinea pigs on days 30 and 60 (mean+SD, n = 5). The stars denote statistically significant increase in NAC-treated animals compared to the control animals (* = p<0.05 and ** = p<0.01).

There was a statistically significant increase in nuclear expression of Nrf2 (p<0.05) within lung primary granulomas on days 30 and 60 in the NAC-treated group compared to mock-treated control groups (Figures 9A and 9B). Moreover there was a statistically significant increase in NQO1 expression (p<0.05) within primary granulomas on days 30 and 60 in NAC-treated guinea pig compared to the mock-treated control groups (Figures 9C).

An increase in whole blood reduced-glutathione (GSH) was seen in NAC-treated animals compared to the mock-treated control group on day 60 of infection (p<0.05). No significant differences were noted in GSH levels on day 30 of the infection and in oxidized-glutathione (GSSG) levels between the control and NAC-treated animals throughout the course of infection. Similarly increases in the GSH/GSSG ratio was not statistically significant in NAC-treated animals compared to the control group on day 30 and but increased significantly by day 60 of infection (p<0.005) in NAC-treated animals (Figure 9F). The total antioxidant capacity of the serum was statistically significant increase (p<0.05) in NAC-treated guinea pigs compared to mock-treated controls by day 60 of infection (Figure 9G).

## Discussion

The clinical manifestations of tuberculosis in humans and animals are directly proportional to the severity and extent of pulmonary and extra-pulmonary inflammation [24]. In the early stages of *M. tuberculosis* infection in humans lesions are composed primarily of macrophages and granulocytes with fewer lymphocytes [4]. Similar cell phenotypes are seen in the early stages of experimental *M. tuberculosis* infection in guinea pigs. The significance of these findings is that like in other species, macrophages and granulocytes are among the most potent generators of oxygen free radicals during an inflammatory response [14], [25], [26]. Previously, we showed that the development of lesion necrosis correlated with an increased influx of macrophages and granulocytes in the lung and that could be prevented in guinea pigs that were vaccinated with BCG prior to *M. tuberculosis* challenge [14], [25]. Besides co-localizing to sites of necrosis, we showed that these inflammatory cells released their cytoplasmic contents resulting in extracellular accumulation of iron and copper, both of which catalyze free radical generation [25], [27].

Our primary objective in this study was to establish the presence of oxidative stress conditions during experimental tuberculosis in guinea pigs and to determine whether antioxidant therapy could reverse the adverse effects of progressive inflammation in this model. Since reactive oxygen and nitrogen species have extremely short half-lives and are difficult to measure directly, we evaluated the byproducts of free radical generation *in vivo* and measured the serum and tissue antioxidant capacity of *M. tuberculosis* infected guinea pigs over time. The oxidative stress marker, MDA, increased in lung lesions as *M. tuberculosis* infection in guinea pigs progressed. Interestingly besides macrophages and granulocytes, vascular endothelial cells associated with lesions also showed prominent MDA staining as the infection progressed. The significance of this finding is that free radical-mediated endothelial damage may contribute to the pathogenesis of lesion necrosis by promoting microvascular thrombosis leading to lesion ischemia [28].

The serum total antioxidant capacity was significantly depleted in *M. tuberculosis* infected guinea pigs as early as 15 days of infection. These data confirmed that oxidative stress conditions occur in the early stages of infection and is established systemically even before lesions are advanced. BCG vaccination prior to aerosol challenge maintained the serum antioxidant capacity in *M. tuberculosis* infected guinea pigs near the levels of un-infected control animals. However, the serum antioxidant capacity decreased even in BCG-vaccinated animals by day 60 of the infection is consistent with the loss of immune protection conferred by vaccination in the chronic stages of disease [14].

As glutathione is among the most important intracellular and extracellular antioxidants, we compared the total, reduced- and oxidized-glutathione levels in whole blood of *M. tuberculosis*-infected guinea pigs. Reduced-glutathione (GSH) levels were markedly decreased as the infection progressed which was reflected by a significant decrease in the GSH/GSSG ratio. These data are consistent with the depletion of total antioxidant capacity, which is due in part to total, and more specifically reduced blood glutathione levels. Despite the decrease in glutathione levels, an overall increase in total glutathione expression in lung lesions was indicative of the host response to oxidative stress as a consequence of *M. tuberculosis* infection. Since the anti-glutathione antibody used in this study does not distinguish between the reduced and oxidized forms, immunohistochemistry cannot be used to evaluate the relative antioxidant capacity due to glutathione within lesions using this method. The MDA expression in lung lesions combined with the decrease in serum total antioxidant capacity, and GSH/GSSG ratio, confirms the presence of oxidative stress conditions in experimental tuberculosis in the guinea pigs similar to what is seen in the naturally occurring human disease [6], [8].

Our second objective was to identify potential mechanisms to explain the failure to maintain adequate antioxidant capacity in *M. tuberculosis* infected guinea pigs. Since the redox sensitive transcription factor Nrf2 regulates the expression of several important antioxidant proteins and nuclear translocation is necessary for normal Nrf2 function, we evaluated the relative nuclear to cytoplasmic expression in lung lesions and primary lesion free lung using immunohistochemistry. Overall Nrf2 expression increased in primary lesions as the infection progressed in non-treated and non-vaccinated animals. This increased expression correlated significantly with the progression of disease that was reflected in the increased lesion pathology scores. Despite the increase in cytoplasmic Nrf2 expression, there was a significant lack of nuclear staining in primary lesions compared to naïve or non-affected regions of lung parenchyma. The lack of nuclear expression of Nrf2 within primary granulomas also correlated with the lack of expression of the Nrf2-regulated antioxidant protein NQO1 within the primary granulomas. NQO1 expression was limited to the areas that had Nrf2 nuclear expression (non-affected lung regions), and was decreased within primary granulomas that lacked nuclear expression. Another known Nrf2-regulated enzyme GCS did not follow a similar pattern. In fact, GCS had moderately high level of expression throughout the course of the infection with minimal change in expression levels between different time points and between primary granulomas and primary lesion free lung parenchyma. This is consistent with previous studies that showed that GCS expression is also regulated by Nrf2-independent mechanisms [29], [30].

A similar lack of nuclear translocation of Nrf2 has been previously reported in an *in-vitro* model of cigarette smoke-mediated oxidative stress. In that study, treatment with an Nrf2-inducing drug, resveratrol, induced nuclear translocation of Nrf2 and subsequent quenching of cigarette smoke generated free radical release [31]. The cytoskeleton serves as scaffolding to bind and regulate nuclear translocation of Nrf2 within the cell cytoplasm [32]. One possible explanation is that *M. tuberculosis* infection may interfere with this Nrf2 regulatory mechanism directly by altering actin polymerization [33], [34] or indirectly by promoting oxidation of intra-cytoplasmic proteins during infection [35].

Since BCG vaccination prevented the reduction in systemic antioxidant capacity in early stages of the infection, we determined whether the beneficial effects of BCG vaccination prevented the a defect in Nrf2 nuclear expression. However, there was no difference between vaccinated and unvaccinated guinea pigs in the extent of NQO1 and nuclear Nrf2 expression (Figure S3). These data suggest that despite the ability to maintain systemic antioxidant capacity, BCG vaccination fails to prevent the localized defects in antioxidant defenses and thus only delays the manifestations of progressive lung and disseminated disease that ultimately results in death in the guinea pig model [14].

Therapeutic intervention with NAC aimed at reversing defective Nrf2-mediated antioxidant defenses resulted in a decrease in pulmonary and extra-pulmonary lesion and bacterial burden and reduced the severity of lesion necrosis. The improved outcome in NAC-treated animals was associated with increased Nrf2 nuclear staining, granuloma NQO1 expression, increased glutathione (reduced form) levels and improved serum antioxidant capacity compared to the mock-treated controls. NAC serves as an additional source of the amino acid cysteine that is needed for glutathione synthesis but also activates Nrf2 expression, both of which likely contribute to combating oxidative stress during *M. tuberculosis* infection [36]. Based on these data, NAC was effective at delaying the dissemination of bacilli to the spleen suggesting a protective effect on lung vasculature. This response is consistent with the role lesion necrosis and the loss of vascular integrity has in the pathogenesis of extra-pulmonary dissemination of bacilli.

In summary, we have provided evidence that oxidative stress conditions exist in the guinea pig model of tuberculosis similar to what is seen in humans. We also show that systemic and tissue oxidative stress is progressive and correlates with the loss of Nrf2-mediated antioxidant defense mechanisms. Considering the importance of lung lesion progression and the extra-pulmonary dissemination of bacilli in tuberculosis pathogenesis, treating with antioxidant drugs like NAC could be beneficial as an adjunct to conventional anti-tuberculosis drug therapy. An added benefit of antioxidant treatment of patients with tuberculosis is the reduction of the toxic side effects of anti-tuberculosis drug therapy which as also be shown to be mediated oxygen free radical generation in the liver [37].

## Supporting Information

Figure S1 **Negative control staining for antibodies used in this study.** The photomicrographs A, B, C and D represent MDA, glutathione, Nrf2 and NQO1 immunostaining respectively in lungs of *M. tuberculosis*-infected animals either after the addition of antigens in excess to the primary antibody prior to staining for Nrf2 and or after primary antibody being replaced with species-specific polyclonal IgG for glutathione and MDA. As expected no immunostaining in detected in lungs for all four antibodies.
(TIF)

Figure S2 **Nrf2 immunoflourescence in BAL cells after** ***M. tuberculosis*** **infection.** The panels above show Nrf2 protein levels at (A) freshly collected non-cultured; (B) 24 hrs after culture (0 hrs after infection with H37Rv); (C, D, E and F) 4, 24, 48 and 72 hrs after infection with *M. tuberculosis*. Even though both nuclear and cytoplasmic expression of Nrf2 is detected at 24 hrs after activation with phorbol 12-myristate-13-acetate (PMA), only a weak cytoplasmic and no nuclear staining of Nrf2 is noted starting as early as 4 hours after initial infection.
(TIF)

Figure S3 **BCG vaccination fails to alter defective Nrf2 response in infected animals.** The graphs A, B and C represent Nrf2 overall, Nrf2 primary granuloma and NQO1 overall lung immunostaining scores respectively at different time points in BCG-vaccinated *M. tuberculosis*-infected animals. The bars represent median values for each group (n = 5).
(TIF)
