# Supplementary figures and images for: Evidence for Oxidative Stress and Defective Antioxidant Response in Guinea Pigs with Tuberculosis
Source: PLoS One. 2011 Oct 18;6(10):e26254. doi: 10.1371/journal.pone.0026254 (PMC3196542; doi:10.1371/journal.pone.0026254)

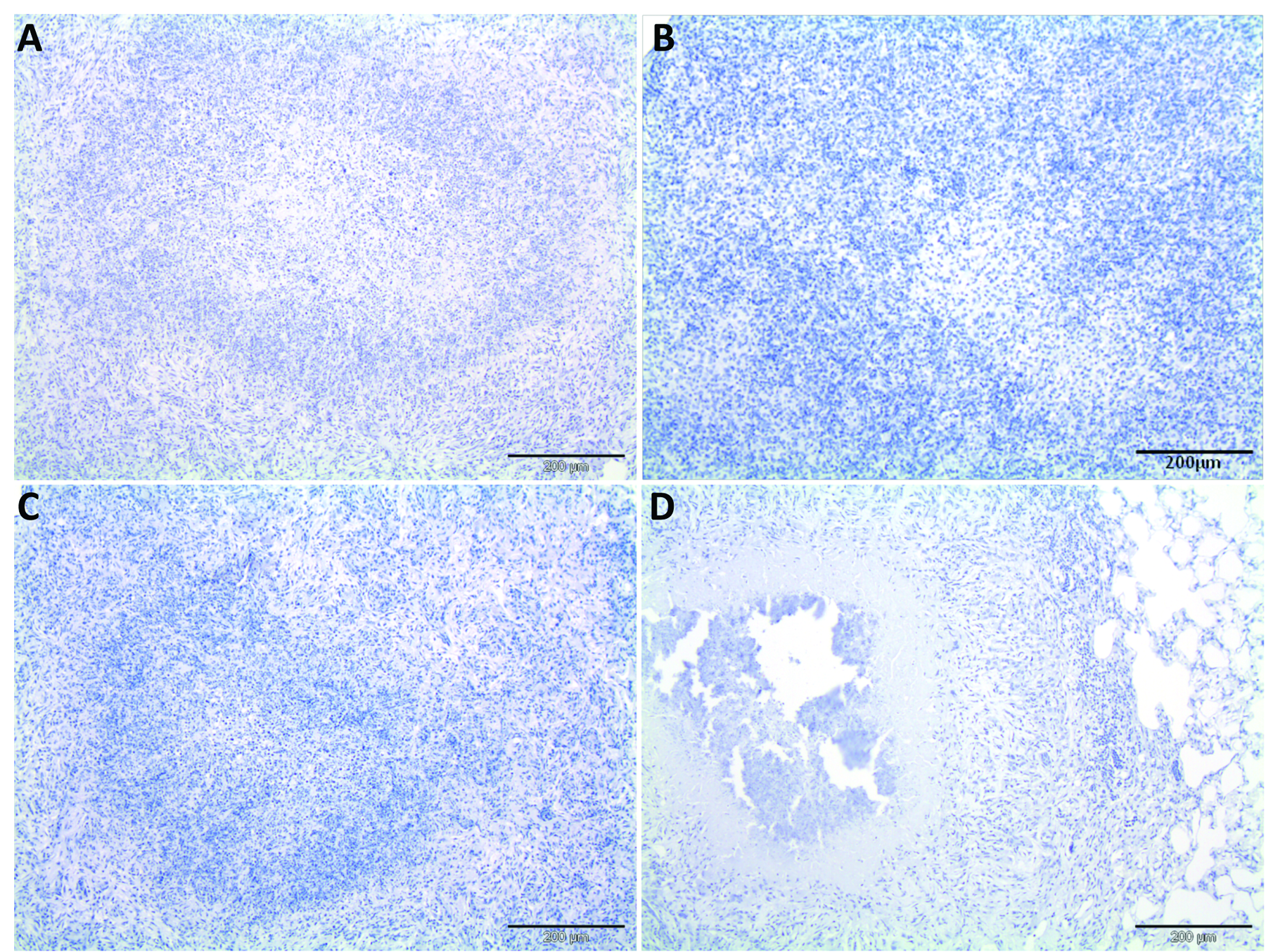

Supplement: Figure S1 — Negative control staining for antibodies used in this study. The photomicrographs A, B, C and D represent MDA, glutathione, Nrf2 and NQO1 immunostaining respectively in lungs of M. tuberculosis-infected animals either after the addition of antigens in excess to the primary antibody prior to staining for Nrf2 and or after primary antibody being replaced with species-specific polyclonal IgG for glutathione and MDA. As expected no immunostaining in detected in lungs for all four antibodies. (TIF) [file pone.0026254.s001.tif]

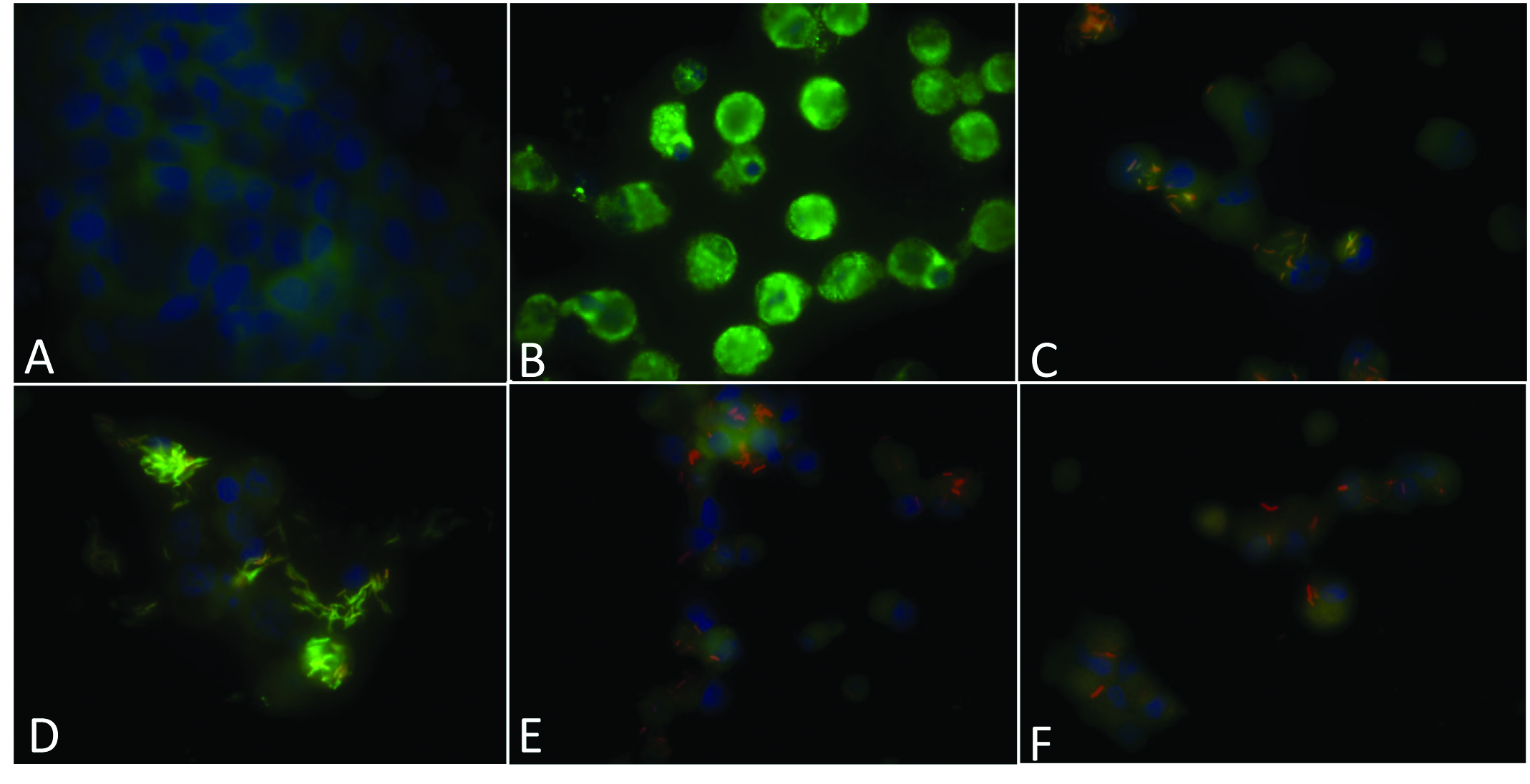

Supplement: Figure S2 — Nrf2 immunoflourescence in BAL cells after M. tuberculosis infection. The panels above show Nrf2 protein levels at (A) freshly collected non-cultured; (B) 24 hrs after culture (0 hrs after infection with H37Rv); (C, D, E and F) 4, 24, 48 and 72 hrs after infection with M. tuberculosis. Even though both nuclear and cytoplasmic expression of Nrf2 is detected at 24 hrs after activation with phorbol 12-myristate-13-acetate (PMA), only a weak cytoplasmic and no nuclear staining of Nrf2 is noted starting as early as 4 hours after initial infection. (TIF) [file pone.0026254.s002.tif]

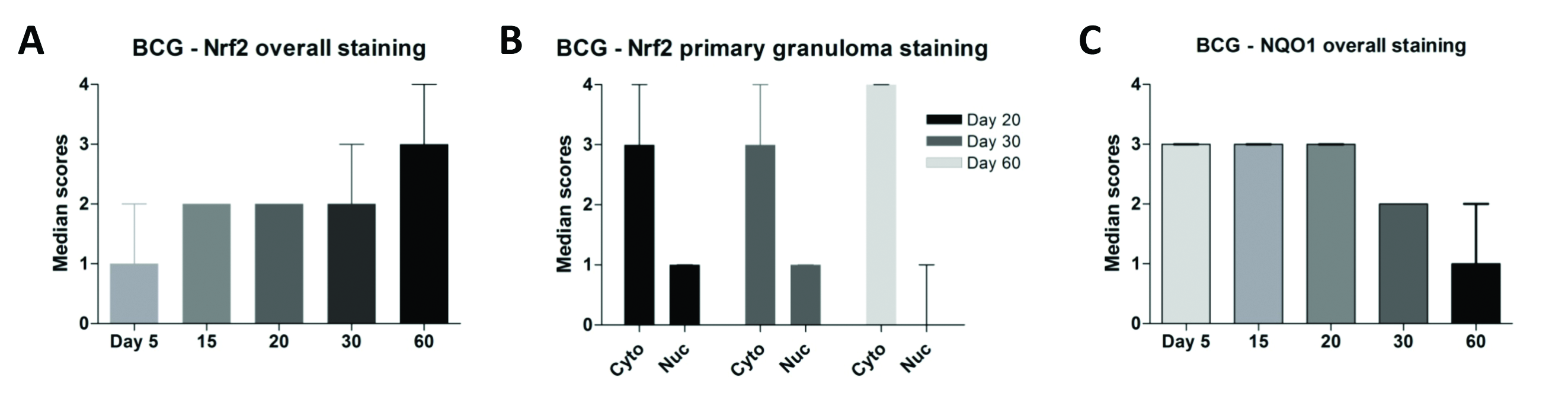

Supplement: Figure S3 — BCG vaccination fails to alter defective Nrf2 response in infected animals. The graphs A, B and C represent Nrf2 overall, Nrf2 primary granuloma and NQO1 overall lung immunostaining scores respectively at different time points in BCG-vaccinated M. tuberculosis-infected animals. The bars represent median values for each group (n = 5). (TIF) [file pone.0026254.s003.tif]
